# Cerebral metastasis of Merkel cell carcinoma following resection with negative margins and adjuvant external beam radiation: a case report

**DOI:** 10.1186/s13256-021-02690-z

**Published:** 2021-03-14

**Authors:** Alex F. Grubb, Elizabeth Hankollari

**Affiliations:** Department of Medicine, Duke University Hospital, 2301 Erwin Road, Durham, NC 27710 USA

**Keywords:** Merkel cell carcinoma, Cerebral metastasis, Immunotherapy, Case report

## Abstract

**Background:**

Merkel cell carcinoma (MCC) is a rare and aggressive neuroendocrine tumor of the skin. It is associated with advanced age, ultraviolet (UV) radiation, and Merkel cell polyomavirus. It has a predilection for the lymphatic system, but rarely spreads to the central nervous system.

**Case presentation:**

A 71-year-old Caucasian man with a history of rheumatoid arthritis and MCC of the right lower eyelid and cheek presented with left-sided hemineglect and word-finding difficulty. Twenty months earlier he had undergone local excision of a 3 cm lesion with negative margins, negative sentinel lymph node biopsy, and external beam radiation. On presentation he was found to have a 6.3 cm mass in the right frontotemporal region. He underwent prompt resection, with pathological analysis consistent with metastatic MCC. He subsequently underwent stereotactic radiosurgery (SRS) and adjunctive immunotherapy with pembrolizumab. He has since tolerated the therapy well and is currently without neurological symptoms or evidence of recurrence.

**Conclusions:**

Cerebral metastasis of MCC is a rare event and should be considered when a patient with a history of MCC presents with neurological symptoms. Optimal treatment regimens of these rare cases are unclear; however, prompt resection, stereotactic radiosurgery, and adjunctive immunotherapy have shown an initial positive response in this patient.

## Background

Merkel cell carcinoma (MCC) is a rare and aggressive neuroendocrine cancer of the skin. This cancer is named because of the dense neurosecretory granules found in the core of the cell which are reminiscent of Merkel cells, the mechanoreceptors found in the basal layer of the epidermis [1, 2]. While these are assumed to be the cells of origin of this malignancy, there are multiple hypotheses that suggest a possible different cell of origin such as immature totipotent stem cells, dermal fibroblasts, or epidermal keratinocytes [3–5]. Pathogenesis is believed to be related to Merkel cell polyomavirus (MCPyV) and ultraviolet (UV) radiation [6–9]. The incidence of MCC is estimated at 0.7 cases per 100,000 persons and predominantly affects older, lighter-skinned adults [10, 11]. Roughly 70% occur on the head and neck and the upper limbs [9, 12]. Treatment is centered on surgical excision for local disease; however, there is frequent local recurrence [12, 13]. Metastases are common, particularly to the lymph nodes, and sentinel node biopsy is an important aspect of staging [12]. Cerebral metastases are uncommon, with fewer than 20 cases reported in the literature [14–26]. We report here a case of MCC metastasis to the brain nearly 2 years following excision of the primary skin mass with negative margins, negative sentinel nodes, and local radiation.

## Case presentation

**Fig. 1:**
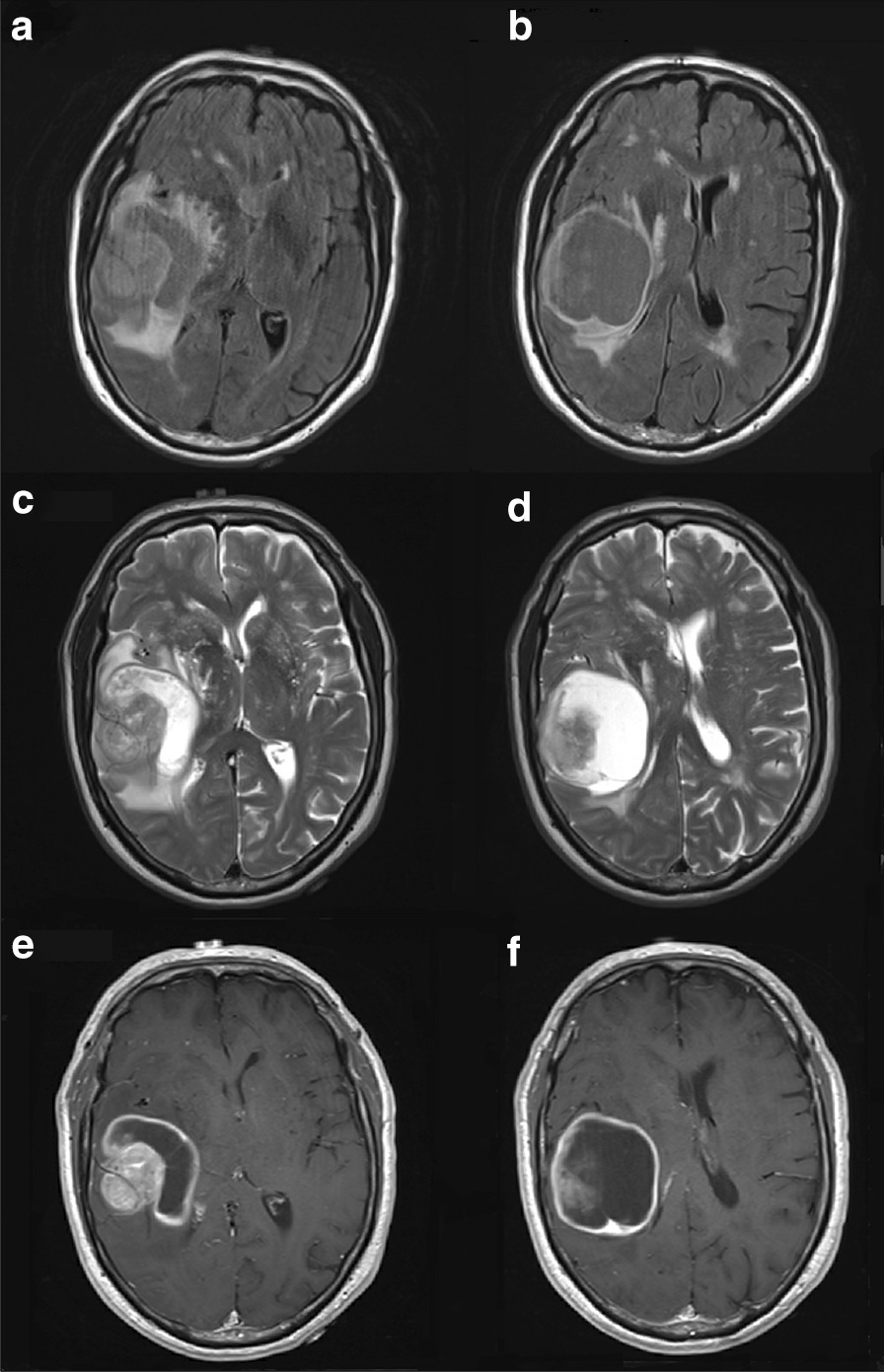
**a**, **b** Fluid-attenuated inversion recovery (FLAIR) and **c**, **d** T2-weighted imaging show increased T2 signal involving the right temporal lobe and right frontal lobe and no evidence of hemorrhage. A 9 mm right-to-left midline shift is shown with early right uncal herniation, effacement of the right lateral ventricle, and dilatation of the temporal horn of the right lateral ventricle. The hyperintense areas surrounding the lateral ventricles (**a**, **b**) are likely representative of leukoaraiosis rather than tumor lesions. **e**, **f** T1 imaging shows a 6.3 cm × 5.5 cm × 4.8 cm ring-enhancing heterogeneous cystic mass in the right frontotemporal region with a 3.5 cm × 3.0 cm avidly enhancing mural nodule with diffusion restriction (diffusion imaging not shown) on the lateral aspect of the mass

**Fig. 2:**
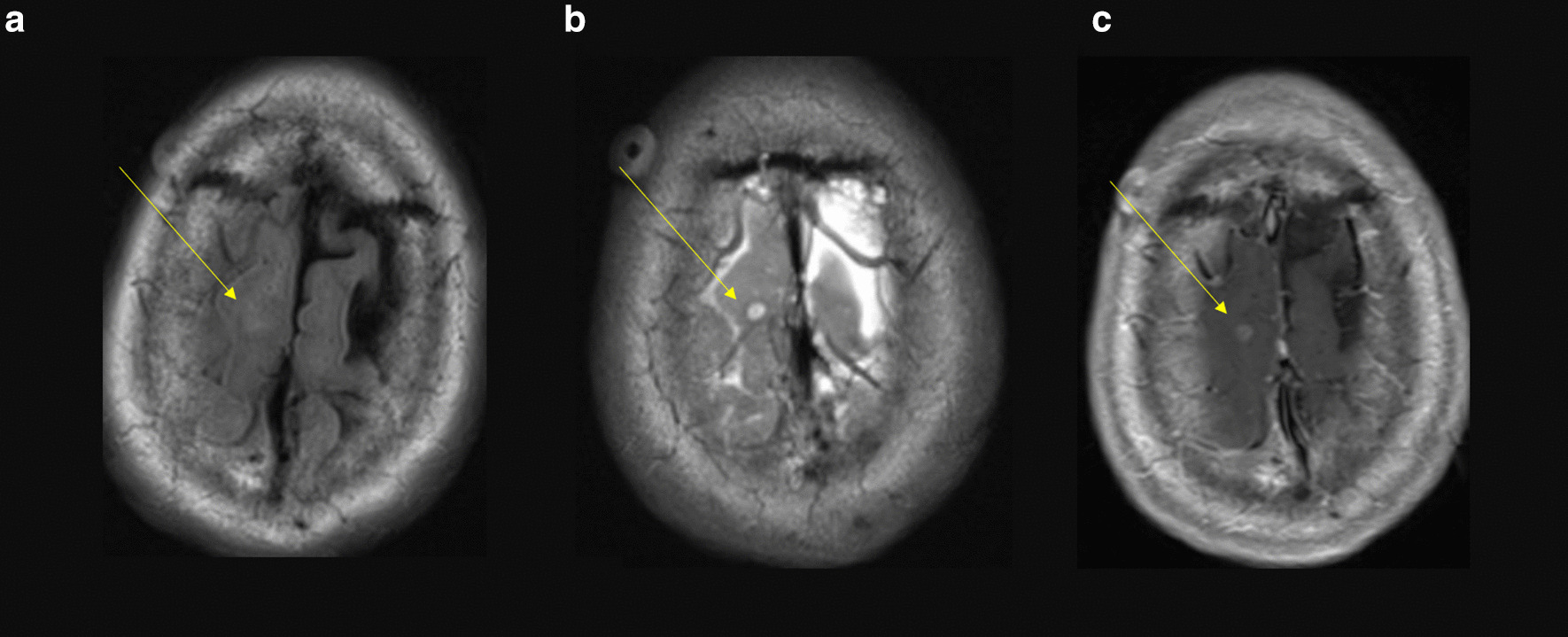
Fluid-attenuated inversion recovery (FLAIR) (**a**), T2 (**b**), and T1 (**c**) images demonstrating a 5 mm enhancing lesion (yellow arrow) in the right superior frontal gyrus

A 71-year-old Caucasian man presented to the emergency department with 3 weeks of forgetfulness, word-finding difficulty, gait disturbance, and left-sided hemineglect. He had a history of a 3 cm × 2 cm T2N0M0 stage IIa MCC of the right lower eyelid and cheek which had been treated with local excision with negative margins, negative sentinel lymph node biopsy, and external beam radiation (45 Gray total) to the parotid and ipsilateral neck roughly 20 months earlier. No imaging of his brain was performed at that time. His past medical history was significant for hypertension and rheumatoid arthritis (RA), for which he was prescribed hydroxychloroquine. Upon presentation to the emergency department he had normal vital signs. His physical exam was notable for left-sided hemineglect as well as left-sided tactile extinction. His laboratory results were notable only for a small increase in his serum creatinine from 1.1 to 1.4*.* Due to a history of anaphylaxis to intravenous contrast, a computed tomography (CT) scan of the brain without contrast was performed, which showed a mass-like area of hypodensity with mass effect on the right lateral ventricle and 6 mm of right-to-left midline shift. Gadolinium-enhanced magnetic resonance imaging (MRI) of the brain (Fig. 1) showed a 6.3 cm heterogeneous cystic and solid mass with surrounding increased T2 signal in the right frontotemporal regions, with an additional 5 mm lesion (Fig. 2) in the middle of the right superior frontal gyrus 9 mm from the longitudinal fissure. A 3.9 mm right-to-left midline shift with trapping of the temporal horn of the right lateral ventricle was described.

**Fig. 3:**
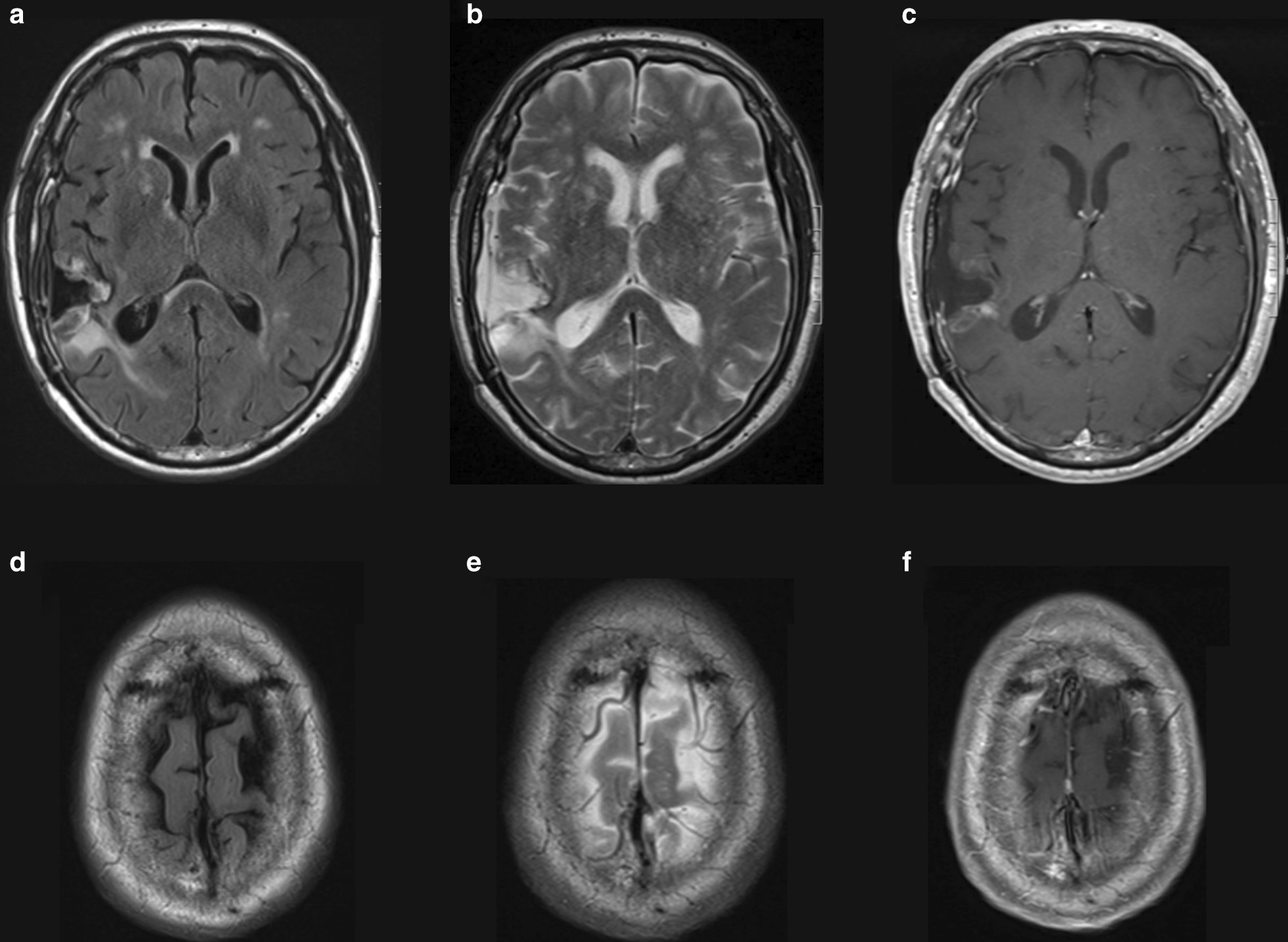
Magnetic resonance imaging of the brain 4.5 months postoperatively. Fluid-attenuated inversion recovery (FLAIR) (**a**), T2 (**b**), and T1 (**c**) show mild irregular rim of enhancement around resection. Resolution of previously seen mass effect and midline shift. Decreased surrounding T2/FLAIR hyperintense signal change. FLAIR (**d**), T2 (**e**), and T1 (**f**) images showing interval resolution of previously noted 5 mm enhancing lesion in the right superior frontal gyrus

Neurosurgery was consulted and the patient was started on dexamethasone and levetiracetam. He was taken to the operating room the following morning for awake craniotomy and resection of the tumor. The neurosurgical team performed a BrainLAB-guided right temporal craniotomy with physiologic monitoring. They removed the entire frontotemporal mass, but did not attempt excision of the 5 mm superior front gyrus lesion. Histological analysis of the tumor showed signs of neuroendocrine features consistent with MCC. Serologic analysis showed he was MCPyV-negative. The patient was discharged home and followed up with radiation oncology and medical oncology. His positron emission tomography (PET)-CT scan showed no evidence of metastatic disease within the chest, abdomen, or pelvis. In order to treat the residual 5 mm right frontal lesion and the resection cavity, he received stereotactic radiation surgery (SRS) to both areas 1 month after diagnosis. SRS was selected over whole brain radiation therapy (WBRT) to avoid the neurotoxic effects from WBRT. He was started on pembrolizumab 1 week after radiation. He experienced improvement in his gait, strength, mental status, and vision in the weeks following treatment. His hemineglect gradually improved over the course of the next 6 months without evidence of new disease (Fig. 3). At 6 months from initiation, he had tolerated pembrolizumab without significant complication or worsening of his RA.

## Discussion

MCC is a rare cancer of the skin which shows a predilection for sun-exposed areas and frequently affects older, lighter-skinned individuals. Regional lymph node metastasis is common at the time of presentation, with roughly 25% of individuals presenting with regional lymph node disease and ~ 10% of individuals with distant lymph node metastases. The 5-year survival rates range from 13 to 55% depending on the size and depth of the initial lesion, presence of occult or clinical regional lymph node metastases, and presence of distant metastases, with the worst prognosis in the last group [12]. Head and neck lesions provide a particularly challenging staging evaluation due to the variable lymphatic drainage in the region [27]. In our patient’s case, it is likely that the sentinel lymph node biopsy was a false negative given his subsequent metastasis.

Central nervous system (CNS) metastases are particularly rare, but have been reported previously in the literature. Fewer than 20 cases have been reported, with variable presentations, survival, and treatment modalities [14–26]. The mechanism for metastasis to the CNS is unclear; however, MCC’s predilection for the lymphatic system and recently discovered lymphatic system of the meninges in humans offers a potential explanation [28]. Due to the rarity of CNS metastases of Merkel cell, the optimal treatment is unknown, but there is some evidence that more aggressive regimens with resection, radiation, and chemotherapy improve survival [14, 20].

Immunotherapy therapy has become the treatment of choice in advanced MCC, with multiple phase I/II trials involving avelumab, nivolumab, and pembrolizumab showing encouraging responses [29–32]. Pertinent to our patient, a phase 2 non-controlled study of pembrolizumab for initial treatment showed a response rate of 56%, with 6-month progression-free survival of 67% [29]. Importantly, this trial showed durable responses, with 85% of responders in remission at 1 year and 79% at 2 years [33]. This was notably superior to traditional chemotherapy regimens, which have shown median progression-free survival of approximately 3 months and disease progression in 90% of patients by 10 months [34]. Avelumab is currently the only US Food and Drug Administration (FDA)-approved immunotherapy for metastatic MCC based on the JAVELIN Merkel 200 study; however, this study notably excluded patients with CNS metastasis and only included patients with previous systemic therapies, thus limiting the applicability to our patient [31].

While adjuvant pembrolizumab is not currently FDA-approved for MCC, the anti-programmed death protein 1 (PD1) agent was chosen based on the size of this MCC lesion (despite radiation therapy), the overall immunogenicity seen in MCC, the efficacy of this therapy in systemic treatment-naïve patients, the efficacy of the therapy in stage III melanoma, and efficacy demonstrated in treating CNS metastases of melanoma [35–37]. There is ongoing research into adjuvant immunotherapy for MCC, with two randomized phase II trials using avelumab or nivolumab. Lastly, given our patient’s history of RA, there is concern for exacerbation in the setting of pembrolizumab use [38]. Fortunately, he has not had exacerbation of his rheumatologic disease to date.

The patient also underwent SRS to his surgical cavity and additional 5 mm lesion. While WBRT is the standard of care for brain metastasis after surgical resection to prevent recurrence, SRS is increasingly used as an alternative [39]. This is because SRS after surgical resection of 1–3 metastases improves survival compared to observation alone and is associated with less neurocognitive decline than WBRT [40, 41]. Still, this is an area of ongoing investigation, and decisions regarding WBRT versus SRS should be made on an individualized basis, with shared decision-making between providers and patient.

This case highlights the aggressive nature of MCC and the difficulty in predicting the clinical course and optimal treatment of these lesions in the head and neck. Despite an initial lesion with negative resection margins and adjuvant radiation, our patient still had subsequent cerebral metastases. Interestingly, our patient had RA and had been treated for years with hydroxychloroquine. MCC incidence is increased among immunosuppressed patients such as those with organ transplantation, B cell malignancies, and human immunodeficiency virus (HIV) [42–44]. While hydroxychloroquine does not “suppress” the immune system in the classical sense, it has many immunomodulatory properties. It is unclear what role, if any, this may have on MCC incidence and progression. This case also highlights a potential blueprint for treatment of these rare metastases with the combination of prompt resection, SRS, and immunotherapy.

## Conclusion

While it is certainly a rare event, consideration should be given for metastatic spread of MCC to the CNS when the patient has a known MCC primary and presents with neurological symptoms. Tissue diagnosis in these cases is of paramount importance, as it will influence treatment regimens moving forward. Aggressive treatment with prompt resection, SRS, and adjunctive immunotherapy has shown favorable results in this patient and should be considered in the rare event of CNS metastasis of MCC.

## Data Availability

Not applicable.

## Abbreviations

MCC: Merkel cell carcinoma
SRS: Stereotactic radiosurgery
MCPyV: Merkel cell polyomavirus
RA: Rheumatoid arthritis
CNS: Central nervous system
PD1: Programmed cell death protein 1
